# Monoclonal antibody therapy of herpes simplex virus: An opportunity to decrease congenital and perinatal infections

**DOI:** 10.3389/fimmu.2022.959603

**Published:** 2022-08-09

**Authors:** Iara M. Backes, David A. Leib, Margaret E. Ackerman

**Affiliations:** ^1^ Department of Microbiology and Immunology, Geisel School of Medicine at Dartmouth, Lebanon, NH, United States; ^2^ Thayer School of Engineering, Dartmouth College, Hanover, NH, United States

**Keywords:** IgG, herpes simplex virus (HSV) infection, monoclonal Ab, neonatal infection, effector function

## Abstract

The fetal/neonatal period represents both a unique window of opportunity for interventions as well as vulnerability to a number of viral infections. While *Herpesviruses* such as herpes simplex virus (HSV) are highly prevalent and typically of little consequence among healthy adults, they are among the most consequential infections of early life. Despite treatment with antiviral drugs, neonatal HSV (nHSV) infections can still result in significant mortality and lifelong neurological morbidity. Fortunately, newborns in our pathogen-rich world inherit some of the protection provided by the maternal immune system in the form of transferred antibodies. Maternal seropositivity, resulting in placental transfer of antibodies capable of neutralizing virus and eliciting the diverse effector functions of the innate immune system are associated with dramatically decreased risk of nHSV. Given this clear epidemiological evidence of reduced risk of infection and its sequelae, we present what is known about the ability of monoclonal antibody therapies to treat or prevent HSV infection and explore how effective antibody-based interventions in conjunction with antiviral therapy might reduce early life mortality and long-term morbidity.

## Introduction

The World Health Organization estimates that around the world over 3.7 billion people have oral herpes infections, and that approximately half a billion people experience genital herpes (1). Herpes simplex virus (HSV) infects the host for their lifetime by infecting neurons of the peripheral nervous system or central nervous system (CNS), the virus can then reactivate asymptomatically or cause cutaneous lesions (2). Whereas current antivirals (acyclovir and its derivatives) decrease the duration and severity of symptoms for millions of adult individuals living with HSV, infections in early life result in substantial morbidity and mortality despite therapy (3–7), therefore adjunct therapy with distinct mechanisms from those employed by small molecule antivirals could provide additive or synergistic benefits. Human and animal model data support that antibodies can provide robust protection in the setting of primary HSV infection. This review will describe the therapeutic prospects of antibody (Ab) mediated protection in primary and recurrent HSV infections and the pipeline to develop monoclonal antibody (mAb) therapy with a focus on neo/perinatal HSV (nHSV) infection.

## Maternal and neonatal HSV infections

### Incidence

**Figure 1:**
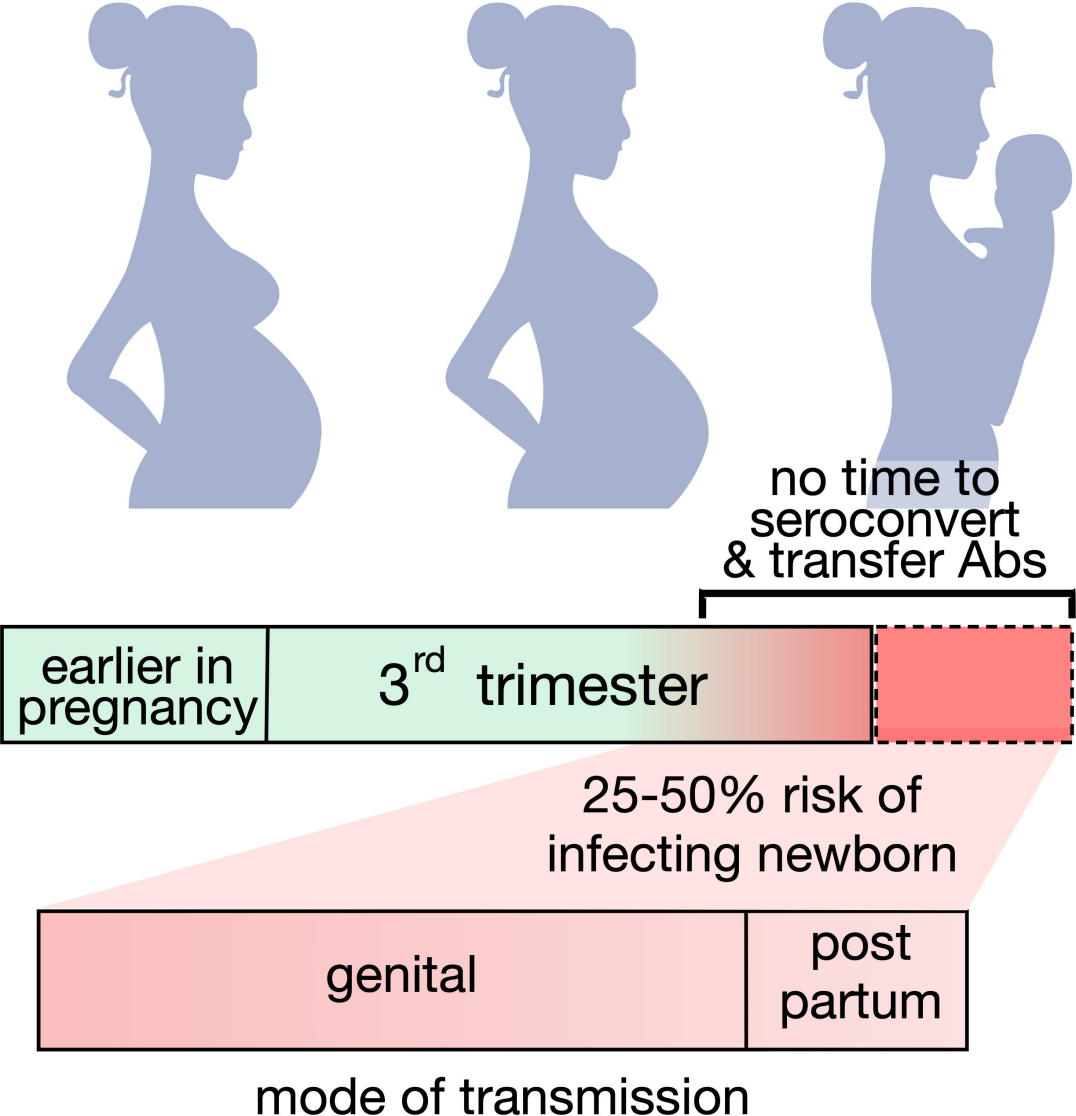
Maternal seroconversion and nHSV infection risk. Graphic representing the potential risk of transmitting neonatal HSV when seroconversion is not achieved or achieved too close to delivery. The majority of infants acquire disease from infected mothers during birth.

Approximately 2 – 4% of women acquire HSV during pregnancy (8). While both HSV serotypes can result in nHSV, HSV-1 predominates in the Americas, Europe and the Western Pacific, and HSV-2 predominates in Africa, South East Asia, and the Eastern Mediterranean (9). Maternal infection during pregnancy is managed with anti-viral therapy and can resolve without severe outcomes in both mother and child (10). Strong epidemiological evidence as to the importance of antibodies in preventing HSV infection comes from the dramatic influence of maternal seropositivity on nHSV risk (**Figure 1**). Primary maternal infections acquired during late gestation present a significant risk (25 - 50%) of transmitting HSV to neonates when compared to women with recurring genital infections (< 3%) (11, 12). The basis for this reduction is believed to be derived from the development and transfer of protective maternal IgG antibodies, which cannot be achieved if infection takes place close to parturition. Thus, whereas an effective HSV vaccine to prevent adult-to adult spread remains a challenging goal (13, 14), accomplishment of nHSV protection, with its defined and short period of risk, may be readily achievable.

### Timing and outcomes

*In utero* infections are rare, representing 5% of nHSV infections. The majority of nHSV infections, 85%, are acquired during parturition, while the remaining 10% of infectious take place post-partum *via* close contact. Most vertically transmitted nHSV results from asymptomatic viral shedding of a pregnant parent, as symptomatic maternal infections often result in birth *via* cesarean section, which significantly reduces the risk of transmission (8, 11, 15).

Congenital HSV infections may result in skin vesicles or scarring, eye involvement, microcephaly, and hydranencephaly that are associated with severe neurological morbidity, and blindness. Vertical transmission of HSV due to placental hematogenous spread of infection, as well as amniotic infection, have both been reported. Despite the low numbers of congenital infections, detection of HSV DNA in the placenta is more common than one would expect. In a recent study, 37% of placentas (n = 160) assessed were positive for HSV-1 viral DNA, with previous reports ranging from 4 – 28% (16), while HSV-2 DNA was detected in 9% of placentas (17). Both studies also reported the presence of HSV DNA in neonatal cord blood, however neither study had appropriate follow up to determine if clinically evident neonatal HSV infections took place following the detection of HSV DNA (16, 17).

**Figure 2:**
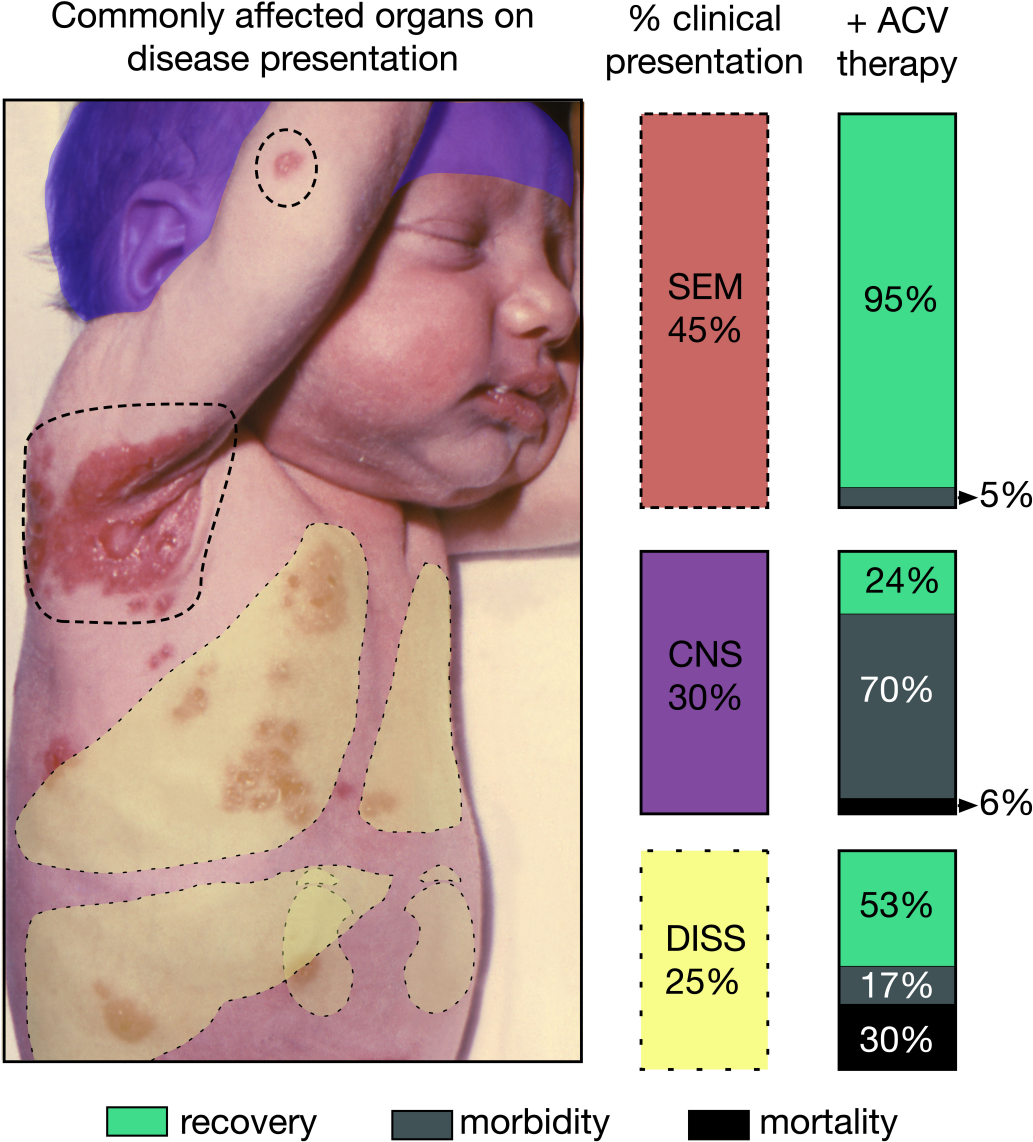
Consequences of neonatal herpes simplex virus (nHSV). Graphic depicting the different presentations and their respective estimated occurrence in the clinical setting. Outcomes of each disease presentation when treated with ACV are also displayed for each presentation to the right of color-coded columns. Skin, eye and mouth (SEM) disease often shows characteristic lesions, some of which are highlighted with dotted lines. Disseminated (DISS) can often affect lungs, liver, kidneys, and adrenals depicted as yellow overlays. Central nervous system (CNS) disease depicted as a purple overlay over affected infant’s head. Infant’s image courtesy of K.L. Herrmann, Centers of Disease Control public image library.

Intrapartum infections tend to be less severe than congenital infections, but can also result in significant morbidity and mortality, often presenting as skin, eye and mouth (SEM) disease, CNS-associated infection, and disseminated viral infection in visceral organs with or without CNS involvement (**Figure 2**). SEM disease typically presents with pathognomonic skin vesicles. If untreated, SEM disease can progress to more severe CNS and disseminated disease. Since the implementation of acyclovir (ACV) therapy and improvements in dosing strategies, more and more cases initially presenting as SEM are resolved before progression to more severe disease (7). However, despite antiviral treatment with ACV, >50% of CNS-associated disease survivors have neurological morbidity, and disseminated disease results in ~40% mortality (7).

Additionally, ACV-resistant viruses are recalcitrant to therapy. The documented mechanisms of ACV resistance involve defects in the virally-encoded thymidine kinase (TK) accounting for ~95% of isolated mutants (18). Generally, TK mutations decrease the fitness of the virus, hindering reactivation and replication (19), thus in the immunocompetent host, TK mutant viruses are rarely isolated (0.3%), and are often expediently cleared (20). For immunocompromised individuals, who rely exclusively on the therapeutic effect of ACV for viral clearance, ACV resistance is a major challenge, and alternatives to ACV are not as readily tolerated and have increased toxicity (18, 20). To date, ACV resistance has not been documented in a neonatal case, but it remains a broader public health issue, especially in bone marrow transplant recipients. Thus, while antiviral therapy has undoubtedly reduced the morbidity and mortality of devastating neonatal infections, additional therapeutic interventions are urgently needed to save and improve the quality of life of infected neonates (4, 12, 21).

## Polyclonal Ab-based protection

### Primary disease

The role of polyclonal Abs (pAbs) in protecting humans from primary HSV infection is better defined than its role in reactivated HSV disease. The most compelling evidence for Ab-mediated protection in the setting of HSV disease is the protection afforded to neonates *via* the placental transfer of antibodies (8, 11, 21–23). Specifically, high titers of neutralizing and antibody-dependent cellular cytotoxicity (ADCC) inducing Abs in mothers and infants are associated with the absence of disseminated nHSV (24).

### Protection across serotypes

This protection, however, can be HSV serotype specific, as HSV-1 seropositive mothers who acquired first time genital HSV-2 infections close to parturition provided less protection against nHSV. On the other hand, infants born to mothers who were already seropositive for genital HSV-2 and acquired a new genital HSV-1 infection were protected (8). It is not completely understood if this is owed to the anatomical site of infection (orolabial vs. genital) or to serotype-specific differences. It has also been noted however, that people who are HSV-1 seropositive often experience a milder or asymptomatic course of genital HSV-2 infection (25).

Further evidence of type specific infection was also observed during the HerpeVac clinical trial for young women (ClinicalTrials.gov Identifier: NCT00057330). This HSV-2 subunit vaccine targeting glycoprotein D (gD) protected women from genital HSV-1 acquisition, though not from HSV-2 (26). Together, clinical studies and observations all support the notion that Abs offer protection from primary infection, and highlight gaps in our knowledge regarding HSV serotype specific differences in protection.

### Recurring or reactivated disease

While passively transferred Abs clearly provide significant protection in early life, it is also clear that the presence of HSV-specific antibodies do not prevent HSV reactivation in seropositive individuals. Individuals with reactivating HSV disease have been noted to have high binding and neutralizing Ab titers, likely due to repeated antigen exposure (27, 28). Serological and functional analysis of seropositive symptomatic and asymptomatic individuals have proposed that Ab specificity (29) or that naturally occurring Fcγ Receptor and Ab polymorphisms (30) may contribute to viral control. Larger follow up studies are necessary to better understand the clinical impact of these observations and to determine how they may relate to nHSV associated with recurrent/reactivated disease.

### In animal models

Preclinical evidence for Ab-mediated protection during primary infection is extensive and has allowed rigorous and well-controlled evaluation of the role of antibodies in affording protection. In agreement with the clinical studies, pre-existing maternal infections (31), or vaccination with live-attenuated (32, 33), and trivalent-subunit (34) vaccines show protection from nHSV disease. Additionally, these studies have established in both animal models and humans that maternally derived HSV-specific Abs can access the nervous system (35), and can decrease neurological behavioral associated with neonatal HSV infection in animal models (32, 36).

Furthermore, administration of purified HSV-specific IgG to pregnant dams is also protective (32). Together, these findings demonstrate that neonatal protection is mediated *via* passive vaccination, specifically through Ab transfer. Similar evidence is available in the context of adult infection, in a guinea pig model of passive vaccination with HSV-antiserum 24 hours post-infection had significant reduction in vaginal lesions, as well as disease reactivation and latent genome copy numbers (37). Abs that depress the spread of infection within the nervous system *via* passive immunization have protected the mouse eye, and skin, and have also restricted the number of affected sensory ganglia (38–40).

## Towards therapeutic application of mAbs

### Lessons from the past

The reliable isolation of monoclonal antibodies (mAbs) from hybridomas (41) has expanded the experimental tool box to better understand and more reliably dissect the role of Abs and their functional properties in HSV infection (**Table 1**). Antibodies recognizing diverse viral glycoproteins and their various subdomains have been isolated and screened *in vitro* for neutralization, complement-dependent cytotoxicity (CDC) or ADCC activity and *in vivo* for preventing infection. While these activities tend to be considered in isolation, they can be synergistic, as demonstrated by the ability of complement to enhance neutralization and ADCC (57, 58). Several important observations relating to protective mAb properties arose from these studies: 1) Different glycoprotein targets could confer protection, however, not all epitopes within or antibodies recognizing the same glycoprotein protected equally. 2) Protection was highly specific, as single point mutant viruses could abolish mAb efficacy. 3) Protection could be derived from neutralization, though most studies reported superior protection *via* ADCC mechanisms. 4) mAbs displayed variability in their protective capacity depending on when and how they and viral challenges were administered.

**Table 1:** Summary of studies investigating the role of antibodies in herpes simplex virus infection. .

mAbs	Ag.	Subtype		Functions	Findings	References
13aC5	gC	IgG1		CDC	0% survival of adult mice challenged *via* f.p with HSV-2	(42)
17aA2	gC	IgG2a		ADCC CDC	70% survival of adult mice challenged *via* f.p with HSV-2	
17bA3	gD			ADCC CDC	75% survival of adult mice challenged *via* f.p with HSV-2	
17bC2	gE	IgG2a		ADCC	35% survival of adult mice challenged *via* f.p with HSV-2	
18aA5	gC	IgG1		ADCC	55% survival of adult mice challenged *via* f.p with HSV-2	
20aD4	gB	IgG1		ADCC CDC	75% survival of adult mice challenged *via* f.p with HSV-2	
HC1	gC	IgG2a		NT, ADCC	Protected adult mice from neurological illness and death w/HSV-1 challenge, but not HSV-2 *via* f.p challenge (1) 86% survival of 1 wk old neonatal mice challenged with HSV i.p. (2)	(1, 43) (2, 44)
HD1	gD	IgG2a		NT, ADCC	Protected adult mice from neurological illness and death after f.p. HSV-1 and HSV-2 challenge (1) 57% survival of 1 wk old neonatal mice challenged with HSV i.p. (2)
IIIE8	gC	IgG2a		No NT	adult mice challenged with HSV-1 intravaginally reduced mortality, skin lesions and ganglionic infections	(45)
HSV 863	gD	IgG1		NT	HSV-1 protective 24 hrs post infection, HSV-2 protective 48 hrs post infection (2)	(1, 15) (2, 46)
HS1	gB	IgG2a		NT, ADCC	Prevents death in adult athymic nude mice challenged intracutaneously with HSV-1, and adult BALB/C mice from HSV-2 i.p challenge (1) Prevents death in adult athymic nude mice challenged i.p with HSV-1 model of before and after viral challenge up to ~4 days post infection (2)	(1, 47) (2, 48)
H336-1	gB	NR		No NT, No ADCC	0% surv. of 1 wk old neonatal mice when co-administered with adult immune cells in i.p HSV-1 challenge	(44)
H157-1	gB	NR		No ADCC	
H1399-6	gB	NR		No ADCC	
H1359-1	gB	NR		No ADCC	
H126-5	gB	NR		NT, ADCC	
3S	gB	NR		NT, low ADCC	12.5% surv. of 1 wk old neonatal mice when co-administered with adult immune cells in i.p HSV-1 challenge
H1396-7	gB	NR	NT, ADCC		57% surv. of 1 wk old neonatal mice when co-administered with adult immune cells in i.p HSV-1 challenge	(44)
4S	gD	NR	NT, ADCC		75% surv. of 1 wk old neonatal mice when co-administered with adult immune cells in i.p HSV-1 challenge
19S	gC	NR	Low NT, ADCC		86% surv. of 1 wk old neonatal mice when co-administered with adult immune cells in i.p HSV-1 challenge
H1394-1	gB	NR	ADCC		100% surv. of 1 wk old neonatal mice when co-administered with adult immune cells in i.p HSV-1 challenge
H1385-12	gB	NR	ADCC		100% surv. of 1 wk old neonatal mice when co-administered with adult immune cells in i.p HSV-1 challenge
B5	gB	IgG3	CDNT		Highest dose results in 60% survival of adult mice challenged i.c. with HSV-1 (1)	(49)
C11	gC	IgG2a	CDNT		Dose dependent survival of adult mice challenged .c. with HSV-1 (1)
C13	gC	IgG2a	CDNT		High-dose survival of adult mice challenged i.c. with HSV-1 (1)
C14	gC	IgG2b	CDNT	
D3	gD	IgG2a	CDNT of HSV 1 & 2	
C15	gC	IgG2a	low CDNT		Dose dependent survival of adult mice challenged .c. with HSV-1 (1)
C16	gC	IgG2a	low CDNT	
B6	gB	IgG2b	CDNT, ADCC		High-dose survival in adult mice from i.c. challenge with HSV-1 (1) 11% of mice sustained zosteriform spread after HSV-1 challenge, protective (2)	(1, 49) (50) (2)
D2	gD	IgG3	med CDNT, med ADCC		High-dose survival in adult mice from i.c. challenge with HSV-1 (1) 63% of mice sustained zosteriform spread after HSV-1 challenge, not protective (2)
B4	gB	IgG3	CDNT, low ADCC		High-dose survival in adult mice from i.c. challenge with HSV-1 (1) 76% of mice sustained zosteriform spread after HSV-1 challenge, not protective (2)
B3	gB	IgG3	low HSV-1 CDNT, ADCC		High-dose survival in adult mice from i.c. challenge with HSV-1 (1) 77% of mice sustained zosteriform spread after HSV-1 challenge, not protective (2)
C3	gC	IgG2a	CDNT, high ADCC		Whole IgG protected against zosteriform spread, Fab fragments did not. Dose dependent survival in adult mice from i.c. challenge with HSV-1 (1) 17% of mice sustained zosteriform spread after HSV-1 challenge, protective (2)
C4	gC	IgG2a	CDNT, high ADCC		Whole IgG protected against zosteriform spread, Fab fragments did not. Protected adult mice from IC challenge with (1) 21% of mice sustained zosteriform spread after HSV-1 challenge, protective (2)	(1, 49) (2, 50)
B8	gB	IgG2a	CDNT, low ADCC		100% of mice sustained zosteriform spread after HSV-1 challenge, not protective (2)	(50)
C8	gC	IgG2a	CDNT, high ADCC		43% of mice sustained zosteriform spread after HSV-1 challenge, somewhat protective (2)
D7	gD	IgG2a	CDNT, high ADCC		7% of mice sustained zosteriform spread after HSV-1 challenge, protective (2)
D8	gD	IgG2a	CDNT, high ADCC		10% of mice sustained zosteriform spread after HSV-1 challenge, protective (2)
H7E	gE	IgG1	low NT, ADCC		Protected adult mice from ocular challenge with HSV-1 at 24 hr pi	(51)
F3AB	gB	IgG1	low NT		Protected adult mice from ocular challenge with HSV-1 at 24 hr pi
G8C	gC	IgG2B	NT, CDNT, ADCC, ADCL		Protected adult mice from ocular challenge with HSV-1 at 4 & 24 hr pi
D8AB	gB	IgG2B	low NT, ADCC		Protected adult mice from ocular challenge with HSV-1 at 4 & 24 hr pi
1S	gD	IgG2a	med NT		Protected adult mice from ocular challenge with HSV-1 at 4 & 24 hr pi
AP7	gD	IgG2a	CDNT		Protected adult mice from zosteriform spread	(52)
LP11	gH	IgG2a	NT	
LP2	gD	IgG2a	NT	
LP3	gD	IgG2a	no NT		Did not protect adult mice from zosteriform spread
8D2	gD	IgG2a	NT HSV 1 & 2		Protects adult mice from stromal keratitis induced by corneal challenge with HSV-1 (1) Protects CD4 or CD8 T-cell depleted adult mice from keratitis and encephalitis when administered 24 hrs pi following corneal challenge with HSV-1 (2)	(1, 53) (2, 54)
CH42	gD	IgG1 (AAA mutation)	no NT, ADCC		Reduced mortality in adult mice when challenged with HSV-1 *via* the cornea, and reduced viral DNA in peripheral nervous system Reduced mortality in i.n. HSV-1 and HSV-2* challenge of neonatal mice *CH43 not tested in HSV-2 challenge.	(1, 55) (2, 56)
CH43	gD	IgG1 (AAA mutation)	no NT, ADCC	

CDNT, complement-dependent neutralization; NT, neutralization; f.p, foot pad; i.p, intraperitoneal; i.c, intracranial; i.n, intranasal; pi, post-infection.

Neutralization continues to be a benchmark for therapeutic efficacy in the setting of diverse viral infections. In the past this therapeutic strategy has proven fruitful, as Palivizumab, a respiratory syncytial virus (RSV)-neutralizing mAb, has been a highly successful intervention in neonates in RSV infection in high risk infants (56, 59). Prophylactically administered Palivizumab (MEDI-493) reduced hospitalization due to RSV disease by 55% when compared to placebo (60). Both direct and indirect antiviral activities can be readily achieved by a single mAb. For several decades, however, the importance of neutralization-independent, Fc-mediated functions have been noted in humans as well as in animal models. A recent review (61) highlighted the protective effect of FcγR-mediated effector functions in protection from adult HSV. Here we will focus on remaining questions regarding the role of Ab Fc in neonates and neonatal models of infection.

The age-dependent susceptibility of neonates to HSV motivates further understanding of Ab-protective mechanisms of action in this specific population. Earlier studies demonstrated that co-infusion of neonatal mice with human IgG, interferon, and immune cells could protect these mice from lethal challenge (62), therefore it was of interest to determine if Abs to specific epitopes and with defined ADCC and neutralizing activities could also protect neonatal mice. While pAbs reactive with gD or gB peptides protected against low-dose viral challenge model, adult mouse macrophages, which are strong mediators of ADCC, were also required to protect against high dose challenge. Similar results were observed for mAbs specific for gB, gC, and gD, which were shown to be protective when co-administered with macrophages and when they had high ADCC activity (44). Therefore, these studies demonstrated that both neutralization and ADCC activity were associated with protection, in agreement with clinical studies in which high neutralizing or ADCC-inducing Ab titers were independently associated with the absence of disseminated disease in neonates (24).

Thus, while these studies supported the notion that ADCC mediated by macrophages is a protective mechanism of action against HSV infection in mice, they also suggested that neonatal immune cells may not be sufficient to elicit a protective response in the setting of a high dose challenge. In the clinic, however, neonates born to mothers with high neutralizing or ADCC antibody titers are protected from disseminated viral disease, suggesting that neonatal immune cells are functionally capable of protecting neonates, or high neutralizing titers can compensate for low ADCC function, which is typically mediated by NK cells in humans, or vice versa. Alternatively, these mothers may shed less virus as a result of their own immunological response. An additional caveat is that the mAbs used in previous animal studies were not sufficiently efficacious to prevent disease in newborn mice. While these remain open questions, recent work suggests that diverse mAbs can protect from infection and sequelae in the neonatal mouse model (63), suggesting that clinical administration of systemic or local biologics could emulate and/or improve upon the observed maternal Ab-based protection seen in naturally infected mothers.

### mAbs in clinical trials to treat HSV disease

Given the significant number of individuals affected by recurrent/reactivating HSV, a number of human mAbs tested for efficacy in preventing HSV disease in small animal models are transitioning from bench to bedside (**Table 2**), and may present new options in the prevention and treatment of nHSV infection. Therapeutic mAbs that recognize conserved epitopes required for viral entry may present a particular advantage as these targets are decoupled from the most common mechanisms of small molecule antiviral resistance, and can act at an earlier point in the infection and replication continuum.

**Table 2:** HSV-specific mAbs in clinical trials.

E317/UB-621	E317 is the original clone of the drug product UB -621. Reduced mortality in i.p. HSV-1 challenge of adult mice. Phase 1 clinical trial for s.c. administration proving safe and well tolerated in healthy volunteers. Reduced mortality in i.n. HSV-1 and HSV-2 challenge of neonatal mice. Phase 2 clinical trials for the treatment of recurring genital HSV approved in the US and China (NCT02346760, NCT03595995, NCT04714060, NCT04979975)	(64) (65) WO2010087813A1 (56) (66) US8431118B2 (67)
HSV8	Reduced mortality in i.p. HSV-1 challenge of adult mice. Reduced mortality in i.n. HSV-1 and HSV-2 challenge of neonatal mice. Completed phase 1 trial: Vaginal antibody safety trial: Safety study of monoclonal antibodies to reduce he vaginal transmission of HSV and HIV (NCT02579083)	(68) (56) (69)
2C	Humanized murine mAb, eventually humanized. Protection from viral shedding and mortality in intravaginal and ocular challenge in a mouse model challenged with acyclovir resistant HSV-1. Biological identifier HDIT101 for clinical trials. A topical preparation of h2c will be tested for efficacy in preventing orolabial lesions in participants infected with HSV-1 (NCT04539483), while prevention of anogenital lesions due to HSV-2 infections will also be assessed *via* intravenous infusion (NCT04165122).	(45, 70)

i.p., intraperitoneal; s.c., subcutaneous; i.n., intranasal.

#### 2c

2c targets gB and was isolated from mice immunized with HSV-1 strain 342 hv (45). This mAb binds a discontinuous epitope in domain I of gB (71) necessary for infectivity (72) and is able to neutralize virus with or without complement, and can carry out ADCC. In 1991, Eis-Hubinger et al. described the efficacy of mAb 2c in preventing viral shedding at mucous membranes after intravaginal challenge, and protection from subsequent viral spread to neural tissues and death in C57B6 mice (45). This mAb was later humanized and assessed for efficacy in preventing disease with drug resistant HSV-1 in NOD/SCID mice (70). Humanized 2c (h2c) is able to prevent mortality following intravaginal challenge with a multidrug resistant HSV-1 clinical strain (70, 71), and also protected mice from developing herpetic stromal keratitis in a corneal scarification model (70, 73). The h2c mAb may present an important therapeutic option for immunocompromised patients who are at high risk if infected with resistant strains. Clinical trials in Germany and the United States are currently underway with this mAb under the biological identifier HDIT101. A topical preparation of h2c will be tested for efficacy in preventing orolabial lesions in participants infected with HSV-1 (NCT04539483), while prevention of anogenital lesions due to HSV-2 infections will also be assessed *via* intravenous infusion (NCT04165122).

#### HSV8

HSV8 is a glycoprotein D-specific human IgG1 mAb isolated from an Ab library *via* phage display, with the capability of neutralizing HSV-1 and HSV-2 (74). Topical application of HSV8 prevented genital HSV disease, and systemic delivery prevented death in athymic nude mice challenged *via* the cornea or flank model (68, 75). When added to human cervicovaginal mucus, HSV8 is able to trap HSV, limiting viral movement, presenting an exciting additional effector property to be explored in the setting of sexually transmitted infections (76). In human clinical trials, this mAb has been assessed for local delivery *via* the MB66 vaginal film, in which HSV8 is co-delivered with a broadly neutralizing HIV-specific antibody. The film was well tolerated and allowed for local biologically functional concentrations of mAbs as tested *in vitro* (69). In addition, this mAb protects two-day old mice from HSV-1 and HSV-2 induced mortality (63).

#### UB-621/E317

*E317* is a gD-specific human IgG1 mAb isolated from a single chain variable fragment (scFv) library *via* phage display that can neutralize HSV-1 and HVS-2 (66). The crystal structure of the E317 Fab binding gD has been solved, and this mAb is able to disrupt gD interactions with both Nectin and HVEM receptors (64). Systemic administration of E317 can protect adult SCID mice from lethal viral infection with HSV-1 when administered before (100% survival) or after infection (75% survival) (66). The clinical grade product of this antibody, UB-621, is currently in clinical trials for prevention of orolabial and genital disease (NCT02346760, NCT03595995, NCT04714060, NCT04979975). UB-621 has shown to be protective from HSV-1 and HSV-2 induced mortality in a neonatal mouse model of infection (63).

## Limitations

Barriers to the development and use of mAbs are cost, storage requirements, and route of administration of the drug. While convenient subcutaneous mAb administration is becoming more common, and consumer prices for mAbs vary widely depending on application (77), distribution in resource-limited settings is likely to be highly challenging. Additionally, the timely identification of infected neonates or at-risk pregnancies poses challenges to successful practical deployment based on limited maternal testing. Delays in initiating ACV therapy increase the risk of in-hospital death (78), therefore it is likely that early and/or prophylactic mAb administration would have the best therapeutic outcomes. It is estimated that 3849 women need to be screened in order to prevent one nHSV case that results in severe morbidity or mortality. While screening was effective in reducing the rate of HSV transmission, and also reduced the number of cesarean deliveries, the associated costs were significant (79). We remain optimistic that decreased costs in emerging testing platforms could, in the future, decrease the barriers to implementation of maternal HSV screening.

## Conclusions

The significant mortality and morbidity observed in nHSV infection despite small molecule antiviral therapy demonstrates a critical clinical need for new interventions. Whereas long-term prevention of recurrent reactivation or initial infection in adult populations remains a challenge to modern vaccine development efforts, rich preclinical and epidemiological evidence supports the potential value of antibodies in prevention and treatment of nHSV infection. With prevention of RSV infection *via* palivizumab serving as a model of effective early life antibody therapy and several HSV-specific mAbs in clinical development for adult populations, the evidence reviewed here provides a strong scientific rationale to assess mAbs in human clinical trials for nHSV. Two out of three HSV-specific mAbs in human clinical trials have been tested and shown protection in a neonatal mouse of model of HSV-1 and HSV-2 infection, further bolstering the promise of this approach. Trials for rare diseases, such as nHSV, represent a considerable but worthwhile effort, especially to reduce not just the mortality, but the significant morbidity associated with this devastating neonatal infection.

## Author contributions

IB wrote the initial draft and all authors contributed to manuscript revision. All authors contributed to the article and approved the submitted version.

## Funding

This review was supported in part by NIH NIAID R21 AI147714, P01 AI098681, R01 09083, and U19AI145825.

## Conflict of interest

Authors IB, DL, and MA have received UB-621 and HSV8 from companies involved in their clinical development and have filed provisional patents related to antibody-based prevention and therapy of nHSV infection.

## Publisher’s note

All claims expressed in this article are solely those of the authors and do not necessarily represent those of their affiliated organizations, or those of the publisher, the editors and the reviewers. Any product that may be evaluated in this article, or claim that may be made by its manufacturer, is not guaranteed or endorsed by the publisher.
